# How cell crowding causes cancer cells to spread

**DOI:** 10.7554/eLife.106768

**Published:** 2025-04-21

**Authors:** Rui Hua, Jean X Jiang

**Affiliations:** 1 Department of Biochemistry and Structural Biology, University of Texas Health Science Center San Antonio United States

**Keywords:** DCIS, mechanotransduction, TRPV4, trafficking, calcium homeostasis, cell volume, Human

## Abstract

Cell crowding causes high-grade breast cancer cells to become more invasive by activating a molecular switch that causes the cells to shrink and spread.

**Related research article** Bu X, Ashby N, Vitali T, Lee S, Gottumukkala A, Yun K, Tabbara S, Latham P, Teal C, Chung I. 2024. Cell crowding activates pro-invasive mechanotransduction pathway in high-grade DCIS via TRPV4 inhibition and cell volume reduction. *eLife* **13**:RP100490. doi: 10.7554/eLife.100490.

Cells must constantly respond and adapt to changes in their environment, such as temperature fluctuations and mechanical stress. These responses include cell growth and migration (Rice et al., 2020; Di et al., 2023). Cancer cells must also respond to their environment, in particular to the high density of nearby cells caused by their rapid proliferation. The mechanical pressure caused by this “cell crowding” can make the cancer cells more likely to spread and invade surrounding tissues, but we do not fully understand the mechanisms driving these processes. Understanding how cancer cells respond to cell crowding, and identifying the signals that make them more invasive in these conditions, is important for developing effective cancer treatments.

Ductal carcinoma in situ (or DCIS for short) is a non-invasive form of breast cancer that is confined to the milk ducts. Although it does not spread to surrounding tissue, if left untreated, it can transition into an invasive form of cancer that does spread (Pinder and Ellis, 2003). Now, in eLife, Inhee Chung and colleagues from George Washington University and Thomas Jefferson High School for Science and Technology – including Xiangning Bu as first author – report new insights into how cell crowding drives migration in certain types of DCIS cells (Bu et al., 2024).

First, Bu et al. demonstrated that DCIS cells categorized as more likely to be aggressive based on their appearance (also known as high-grade cells) become more invasive in crowded conditions. These invasive cells had a smaller volume than healthy cells or lower-grade DCIS cells. Cell volume changes are known to be closely linked to cell stiffness, which affects a cell’s ability to push through and invade surrounding tissues (Guo et al., 2017; Xie et al., 2024).

To investigate the mechanism behind this increased invasiveness, Bu et al. used a technique known as mass spectrometry to examine various proteins in the cells. In high-grade DCIS cells, they observed that cell crowding led to an increase in the number of various proteins on the plasma membrane of the cells. In particular, there was a striking 153-fold increase for a calcium ion channel called TRPV4. Transport proteins, such as ion channels and aquaporins, play a crucial role in the regulation of cell volume by creating osmotic gradients that cause water to move in and out of cells (Jentsch, 2016). TRPV4 is known to be activated by osmotic stress, mechanical stress, heat and certain chemical stimuli (Heller and O’Neil, 2007). This movement of TRPV4 and other ion-channel proteins from the cytoplasm to the plasma membrane, as well as reduced cell volume, could also be triggered by increasing osmotic pressure outside the cell, even in the absence of cell crowding.

Based on the finding that TRPV4 relocation was associated with cell crowding, Bu et al. next studied calcium levels in high-grade DCIS cells exposed to different cell crowding densities. This showed that cell crowding decreases intracellular calcium. Bu et al. next blocked TRPV4 function pharmacologically, finding that this mimicked the effects of cell crowding, promoting cell shrinkage and invasion. On the other hand, activating TRPV4 reversed these changes by increasing cell volume.

These results suggest that cell crowding acts as a mechanical cue that inhibits the action of TRPV4, reducing the transport of calcium ions into the cell and thus causing water to move out of the cell, leading to volume loss. This inhibition causes TRPV4 to move from the cytoplasm to the plasma membrane, to ensure that it is primed to become activated by mechanical stress later (Figure 1). Silencing the TRPV4 gene impaired the ability of cancer cells to respond to mechanical cues, leading to less calcium depletion, smaller volume changes, and reduced mobility.

**Figure 1.**
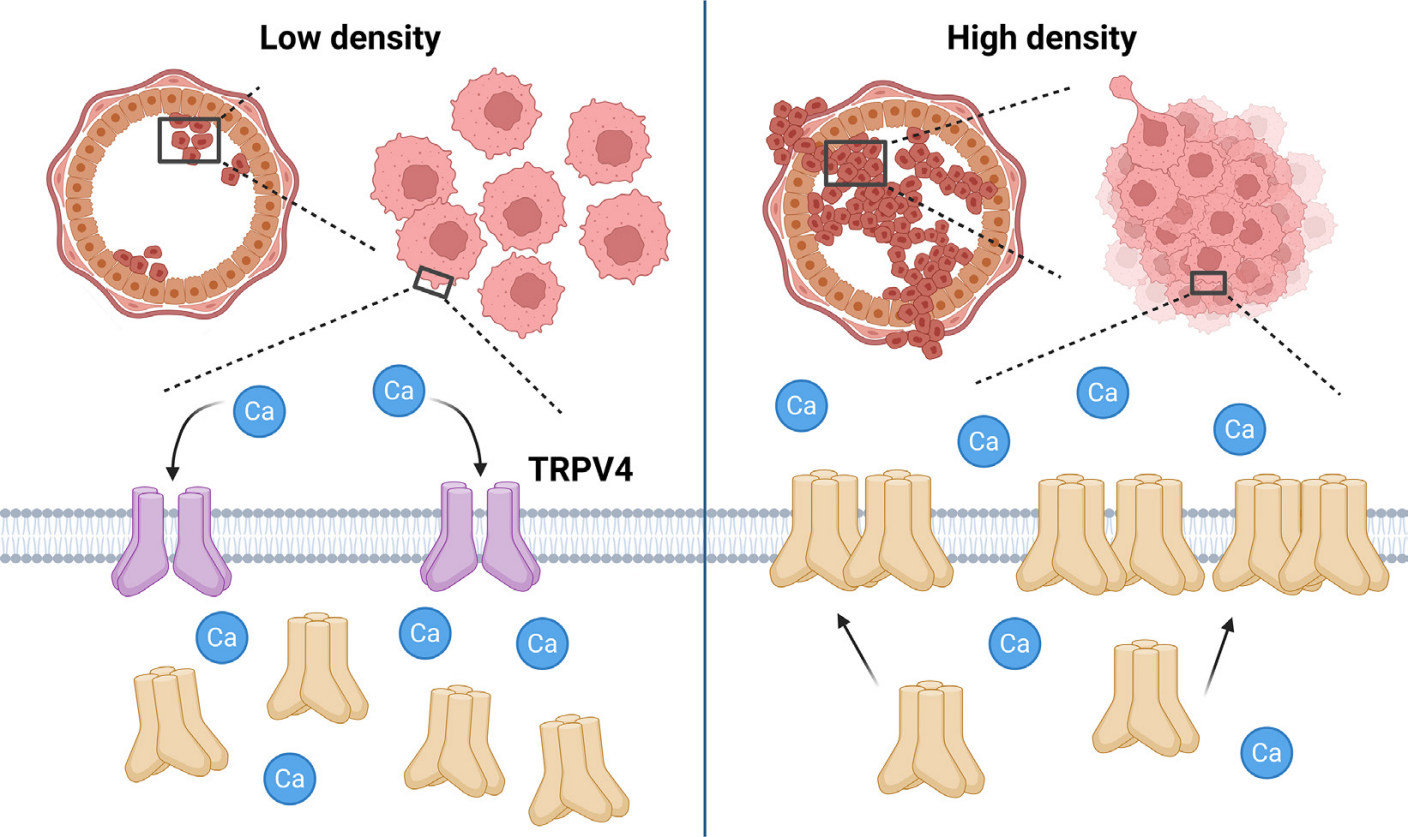
The influence of cell crowding on TRPV4 calcium ion channels. When the density of breast cancer cells (pink) in the milk duct (large circular structure) is low (left), the majority of TRPV4 calcium ion channels are found in the cytoplasm of the cells in an inactive state (cream shapes), with a minority at the plasma membrane (gray) in an active state (purple shapes). The active channels facilitate the movement of calcium ions (Ca; blue circles) into the cell. However, when the density of cells is high (right), the mechanical stress caused by cell crowding inhibits TRPV4 activity and causes TRPV4 to move from the cytoplasm to the plasma membrane (cream shapes). This inhibition of TRPV4 decreases intracellular calcium levels, causing cell volume to shrink, which is a process associated with enhanced cellular invasiveness. The subsequent increase in TRPV4 at the plasma membrane also primes the cell for later activation of TRPV4 to compensate for the loss of calcium ions. These findings suggest that TRPV4 redistribution plays a critical role in the response of cancer cells to mechanical stress, potentially contributing to tumor progression. Created with BioRender.com.

Finally, analyzing patient-derived breast cancer tissue confirmed that TRPV4 is primarily localized to the plasma membrane in high-grade DCIS, but not in lower-grade DCIS or less aggressive cases. These findings suggest that TRPV4 plays a crucial role in influencing crowded cancer cells to become more invasive, as well as serving as a biomarker for identifying high-grade DCIS cells. This could open possibilities for targeting mechanosensitive pathways in cancer treatments and developing diagnostic tools for high-risk DCIS and other cancers.

While the findings of Bu et al. highlight a role for TRPV4 in invasiveness, other ion channels may also be involved (Ranade et al., 2015). Mass spectrometry screening revealed increases in two channel proteins – SCN11 and KCNN4 – caused by cell crowding. Additionally, an ion channel known as PIEZO1 was also found to relocate to the cell membrane under cell crowding conditions. Future research could explore how these channels interact and contribute to cancer adaptation. Other interesting questions include: why do different cell types respond differently to cell crowding, and what molecular mechanisms govern TRPV4 relocation? Addressing these questions could provide deeper insights into how ion channels contribute to cancer progression and help identify potential therapeutic targets.
